# First Insight into the Seroprevalence of Hepatitis E Virus and Associated Risk Factors Among Liver Transplant Recipients from Bulgaria

**DOI:** 10.1089/vbz.2024.0101

**Published:** 2025-04-02

**Authors:** Magdalena Petrova Baymakova, Marina Konaktchieva, Metodi Kunchev, Georgi Popivanov, Todor Kundurzhiev, Ilia Tsachev, Ventsislav Mutafchiyski

**Affiliations:** ^1^ Department of Infectious Diseases, Military Medical Academy, Sofia, Bulgaria.; ^2^ Department of Gastroenterology and Hepatology, Military Medical Academy, Sofia, Bulgaria.; ^3^ Department of Virology, Military Medical Academy, Sofia, Bulgaria.; ^4^ Department of Surgery, Military Medical Academy, Sofia, Bulgaria.; ^5^ Department of Occupational Medicine, Faculty of Public Health, Medical University, Sofia, Bulgaria.; ^6^ Department of Microbiology, Infectious and Parasitic Diseases, Faculty of Veterinary Medicine, Trakia University, Stara Zagora, Bulgaria.

**Keywords:** hepatitis E virus, HEV, liver transplant recipients, liver transplant, seroprevalence, risk factors

## Abstract

**Introduction:**

Hepatitis E virus (HEV) infection is caused by viruses belonging to the *Hepeviridae* family. HEV infection can be self-limiting; however, extrahepatic manifestations may be present. The purpose of the current study was to establish the seroprevalence of HEV among Bulgarian liver transplant recipients (LTRs) and to identify associated risk factors.

**Materials & Methods:**

The present study was conducted between April 1, 2023, and October 30, 2023, at the Military Medical Academy, Sofia, Bulgaria. All serum samples were tested for anti-HEV IgG/IgM using HEV IgG/IgM enzyme-linked immunosorbent assay on Dia.Pro (Milan, Italy). Each participating LTR completed a detailed paper-based closed-ended questionnaire regarding the associated risk factors for HEV infection.

**Results:**

The study included 73 LTRs with a mean age of 47.0 ± 14.0 years. Anti-HEV IgG antibodies were detected in 25 LTRs (34.2%), including 20 males (37.7%) and 5 females (25%). All participants were HEV-IgM negative. HEV seropositivity rates were higher but not statistically significant in LTRs aged >60 years than in those aged <60 years (40% vs. 32.7%). A significant factor by logistic regression was “high level of education” (odds ratio [OR] = 2.917; *p* = 0.038).

**Conclusion:**

To the best of our knowledge, this is the first seroepidemiological HEV study among LTRs from Bulgaria that found a high seroprevalence (34.2%).

## Introduction

Hepatitis E virus (HEV) infection is caused by viruses belonging to the *Hepeviridae* family (ICTV, 2022; Purdy et al., 2022). In 2022, the taxonomy was revised and the family was divided into 2 subfamilies, 5 genera, and 10 species (ICTV, 2022; Purdy et al., 2022). The *Parahepevirinae* subfamily has one genus (*Piscihepevirus*), the members of which infect cutthroat trout (*Oncorhynchus clarkii*) (Batts et al., 2011). The *Orthohepevirinae* subfamily includes four genera (*Avihepevirus*, *Chirohepevirus*, *Paslahepevirus*, and *Rocahepevirus*) and the members infected different mammals and birds (ICTV, 2022; Purdy et al., 2022). The *Paslahepevirus* genus (*Orthohepevirinae* subfamily) has different hosts—humans, camels, cattle, deer, domestic pigs, rabbits, wild boar, etc. (ICTV, 2022). *Paslahepevirus balayani* species (*Paslahepevirus* genus, *Orthohepevirinae* subfamily) has eight genotypes (gt) (ICTV, 2022). HEV gt 1, HEV gt 2, HEV gt 3, HEV gt 4, and HEV gt 7 could induce human infection. HEV gt 1 and HEV gt 2 are anthroponoses with fecal-oral transmission that cause large outbreaks. HEV gt 3 and HEV gt 4 are zoonotic and cause sporadic human infection by eating uncooked or undercooked pig or wild boar meat and meat products and raw deer meat (Tei et al., 2003; Takahashi et al., 2004). HEV gt 3 and/or HEV gt 4 have been found also in *Tursiops truncatus*, cattle, goats, rats, sheep, etc., and a variant of HEV gt 3 has been detected in rabbits (Zhao et al., 2009; Lack et al., 2012; Wu et al., 2015; Di Martino et al., 2016; Huang et al., 2016; Montalvo Villalba et al., 2017). HEV gt 5 and HEV gt 6 have been only isolated in wild boars from Japan (Takahashi et al., 2014). HEV gt 7 and HEV gt 8 have been found in *Camelus dromedarius* from Dubai, United Arab Emirates (Woo et al., 2014) and *Camelus bactrianus* from Xinjiang, China (Woo et al., 2016), respectively. A case of an immunosuppressed patient from the Middle East who regularly consumed camel meat and milk and developed chronic HEV infection (HEV gt 7) has been reported (Lee et al., 2016).

HEV is a nonenveloped, icosahedral, positive-sense, single-stranded RNA with a length of 7.2 kb and has three proteins ORF1, ORF2, and ORF3; furthermore, ORF4 has been found in HEV gt 1 (Koonin et al., 1992; Ding et al., 2017; Montpellier et al., 2018; Yin et al., 2018). The pathogen is transmitted mainly via a fecal-oral route, and zoonotic and blood transfusion transmission exists. Vertical transmission may be realized. The intestinal tract is the initial point of HEV replication before the virus infests the hepatocytes (Yadav and Kenney, 2021). Although the virus has the ability to replicate out of the liver cells (Yadav and Kenney, 2021). Immune mediated cytotoxic T cells and natural killer cells induce liver damage, and nonvirus-specific CD8+ T cells are involved in this process by recent data (Prabhu et al., 2011; El Costa et al., 2021). The interaction between viral factors and host immunity resolves the outcome of the infection (Yadav and Kenney, 2021).

In most cases, the HEV infection is asymptomatic and self-limiting. The main clinical signs and symptoms of acute HEV infection are fever, mild gastrointestinal disorders, fatigue, icterus, loss of appetite, and liver and spleen enlargements (EASL, 2018). Chronic HEV infection (viremia for >3 months) can develop and has been reported in immunosuppressed individuals (Kamar et al., 2008, 2013). Most of these individuals were transplant recipients, but HIV-infected people with rheumatic and hematological disorders receiving heavy immunosuppression were also affected (EASL, 2018).

HEV incidence and seroprevalence among different animal species and human population were widely studied in both developed and developing countries (Songtanin et al., 2023). In Bulgaria, seroprevalence and HEV occurrence were studied widely among animals and humans. Tsachev et al. (2020, 2021a, 2021b, 2023) found the overall HEV seroprevalence to be as follows: pigs, 36.0%; wild boars, 40.8%; East Balkan swine (the only aboriginal pig breed in Bulgaria), 82.5%; dogs, 21.1%; cats, 17.7%; horses, 8.3%; cattle, 7.7%; sheep, 32.2%; and goats, 24.4%. Phylogenetic analysis of acute human HEV cases revealed HEV gt 3*e*, HEV gt 3*f*, and HEV gt 3*c* (Bruni et al., 2018). Different HEV seropositivity (anti-HEV IgG) was observed among different Bulgarian human subpopulations: blood donors, 25.9%; general hunters, 48.7%; hemodialysis patients, 6.2%; HIV-positive individuals, 10.9%; hunters of wild boars, 51.6%; kidney transplant recipients, 8.7%; patients with Guillain-Barre syndrome, 24.5%; and persons with Lyme disease, 12.7% (Baymakova et al., 2021; Golkocheva-Markova et al., 2022, 2023; Kevorkyan et al., 2023).

HEV infection can be dangerous in immunocompromised individuals and is associated with higher mortality. Patients undergoing solid organ transplantation (SOT) are among the high-risk groups of the population. In Bulgaria, this group has not been thoroughly studied for the presence of HEVs. Only a small cohort of kidney transplant recipients was included in a recent study (Golkocheva-Markova et al., 2023). To the best of our knowledge, there are no publications on HEV prevalence among liver transplant recipients (LTRs) in our country. The present study aimed to determine HEV seroprevalence among Bulgarian LTRs and identify the associated risk factors for this infection.

## Materials and Methods

### Study design and data collection

The Military Medical Academy (MMA), Sofia, is the Bulgarian Reference Center for liver transplants (LTs). A prospective database was established on April 21, 2007, when the first LT was performed at our institution. This cross-sectional study used a prospective database. The study was conducted between April 1, 2023, and October 30, 2023, at the Department of Gastroenterology and Hepatology. A list of all the LTs in Bulgaria is presented in Table 1 (EAMS, 2024).

**Table 1.** Dynamics on the Number of Liver Transplants in Bulgaria

Year	LTs in Bulgaria			LTs in MMA		
	Deceased donor	Living donor	Total	Deceased donor	Living donor	Total
2004	0	1	**1**	0	0	**0**
2005	3	5	**8**	0	0	**0**
2006	9	1	**10**	0	0	**0**
2007	6	1	**7**	5	0	**5**
2008	5	4	**9**	4	0	**4**
2009	9	4	**13**	7	1	**8**
2010	13	2	**15**	7	0	**7**
2011	3	3	**6**	2	0	**2**
2012	2	2	**4**	1	1	**2**
2013	7	0	**7**	5	0	**5**
2014	18	1	**19**	10	0	**10**
2015	15	1	**16**	6	0	**6**
2016	10	1	**11**	6	0	**6**
2017	12	1	**13**	7	0	**7**
2018	11	2	**13**	6	0	**6**
2019	11	3	**14**	6	0	**6**
2020	2	5	**7**	0	0	**0**
2021	13	0	**13**	6	0	**6**
2022	6	0	**6**	6	0	**6**
2023^a^	6	0	**6**	6	0	**6**
**Total**	**161**	**37**	**198**	**90**	**2**	**92**

^a^ Until October 30, 2023.

LTs, liver transplants; MMA, Military Medical Academy, Sofia, Bulgaria.

**FIG. 1.**
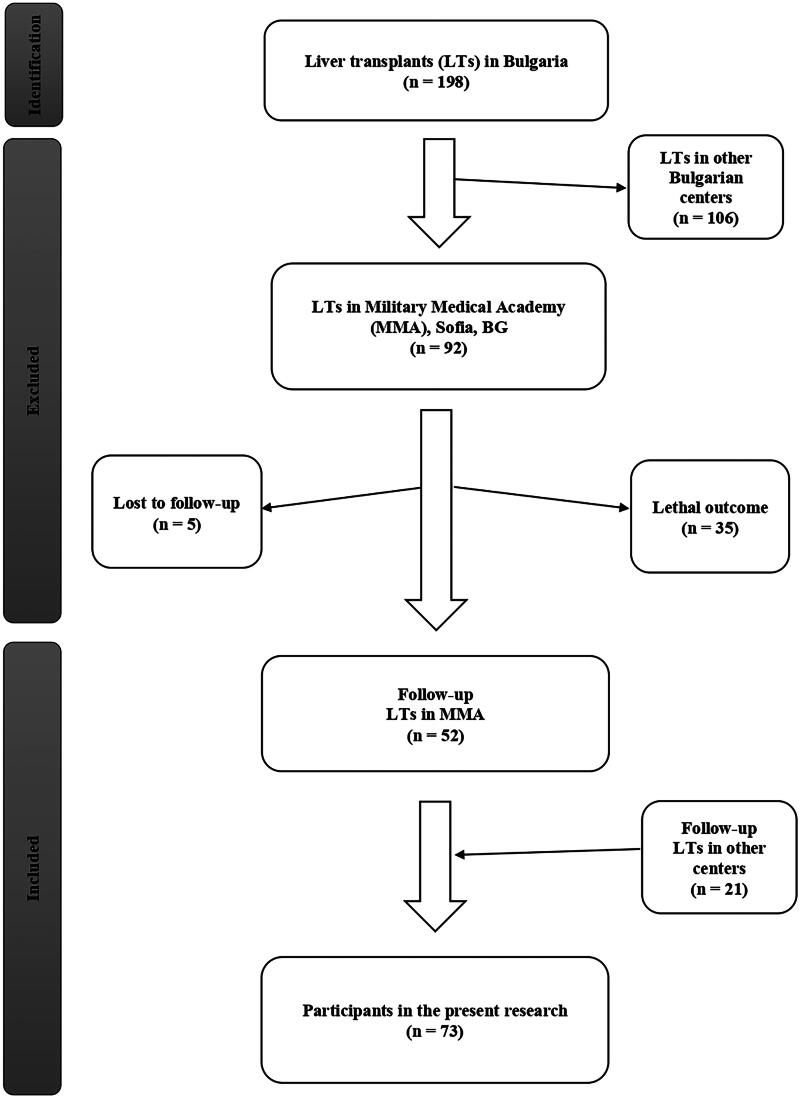
Flow chart showing the selection process of study participants.

Patients with LT who underwent routine annual follow-up at our institution were included in this study (Fig. 1). The follow-up process was guided by a physician (a gastroenterology and hepatology specialist). The following data were collected from each patient: sex, age, place of residence, level of education, household members, tobacco smoking, frequency of alcohol consumption, reason for LT, and immunosuppressive therapy. Tobacco smoking was defined as the regular use of smoked tobacco products. Alcohol consumption was defined as drinking products with a high alcohol content (whiskey, rakia/fruit brandy, etc.) and drinks with a low alcohol content (beer and wine). The follow-up included medical history, physical examination, laboratory investigations, HEV testing, and abdominal ultrasonography. Laboratory indicators included alanine aminotransferase, aspartate aminotransferase, gamma-glutamyl transferase, alkaline phosphatase, total bilirubin, direct bilirubin, total protein, albumin, and creatinine levels. Data were recorded using the hospital’s electronic system (a computer-based software system with limited access).

### Case definition

In this survey, we searched for HEV IgG and IgM antibodies. The presence of anti-HEV IgG positivity was assumed to indicate a history of HEV infection. The presence of anti-HEV IgM positive results was defined as a current HEV infection.

### Inclusion and exclusion criteria

The inclusion criteria for the present survey were as follows: (1) Bulgarian citizens who underwent LT; (2) LTR declared a desire for a follow-up process at MMA; (3) individuals older than 18 years (at the time of HEV testing); (4) written informed consent obtained from each LTR to participate in scientific research; and (5) Department of Virology that has checked HEV status (HEV IgG and HEV IgM antibody testing).

The exclusion criteria for the current study were as follows: (1) foreign citizens who underwent LT (persons without Bulgarian citizenship); (2) individuals younger than 18 years (at the time of HEV testing); (3) persons without written informed consent for participation in a scientific survey; (4) negative results for HEV IgG and/or HEV IgM antibodies; and (5) pregnant women.

### Laboratory investigation for HEV

All 73 samples were subjected to enzyme-linked immunosorbent assay (ELISA) to detect anti-HEV IgG and anti-HEV IgM antibodies. HEV was diagnosed at the Department of Virology, MMA, Sofia, Bulgaria. The following materials were provided by the hospital virology laboratory: calibrated micropipettes and disposable plastic tips; EIA grade water; timer with 60 min range or higher; absorbent paper tissues; calibrated ELISA microplate thermostatic incubator capable of providing a temperature of +37.0°C; calibrated ELISA microplate reader with 450 nm and 620–630 nm filters; calibrated ELISA microplate washer; and vortex or similar mixing tools.

Serum samples (5–7 mL) were collected from each patient. Anti-HEV IgG/IgM ELISA tests were conducted in the Department of Virology according to the manufacturer’s instructions. The collection, transportation, and storage of all serum samples were performed according to the regulatory requirements of MMA and our country.

For the etiological diagnosis of HEV infection, the HEV IgG/IgM ELISA on Dia.Pro (Milan, Italy) was used (Chorami et al., 2021; Frankal et al., 2022). HEV IgG/IgM ELISA tests (Dia.Pro, Milan, Italy) had 100% sensitivity and ≥95% specificity. The borderline results were tested a second time.

### The questionnaire

All study participants completed a detailed and structured questionnaire regarding risk factors associated with HEV infection. The questionnaire was paper-based and closed-ended in nature (Supplementary Table S1). The questionnaire was designed to gather information on consumer behavior (consumption of pork meat, game meat, pork products, seafood, etc.), contact with domestic pigs, activity such as skinning on animals, rodent control, drinking water use, type of sewage, and other factors. The questionnaire was validated by an infectious disease specialist (physician) and a gastroenterology/hepatology specialist (physician). The obtained data formed the associated risk factors for the presence and occurrence of HEV. The present questionnaire was consistent with current knowledge about HEV infection and the experience of other scientists on the current topic (Kmush et al., 2015; Mooij et al., 2018; Capai et al., 2019; Althobaiti et al., 2021; Rajendiran et al., 2024).

### Statistical analysis

Data analysis was performed using Excel 2007 (Microsoft, Redmond, WA, USA) and SPSS Statistics 21.0 (IBM Corp., Armonk, NY, USA). Normally distributed data are presented as the mean ± standard deviation (SD), whereas nonnormally distributed data are presented as the median and interquartile range (IQR). Categorical variables are presented as percentages. A *z*-test was used to test the hypothesis that the observed proportion was equal to the predetermined proportion. Binary logistic regression was used to assess the risk of HEV seropositivity and different basic characteristics/associated risk factors. Statistical significance was set at *p* < 0.05.

### Ethical considerations

All participants provided written informed consent to participate in this study. The LTRs were informed of the entire follow-up process, including the purpose of the survey, confidentiality, procedures, risks/benefits, and participant rights. Imaging and invasive procedures were harmless and painless. Each patient had access to medical records.

Donors for LTs were Bulgarian citizens and citizens of the European Union (EU). All donated organs were obtained with voluntarily full informed consent from donor’s next of kin (deceased donor), or the donor (living donor). LTs were performed according to Bulgarian legislation and all regulations, directives, decisions, recommendations, opinions of the EU.

Scientists participating in this study conducted their activities in accordance with the ethical principles of the Declaration of Helsinki (adopted in June 1964, last revision in October 2013). The present study was approved by the Local Ethics Committee of MMA, 1606 Sofia, Bulgaria (MMA-03/March 09, 2023), which confirmed that the research was in full accordance with all ethical principles and practices. The authors have not used artificial intelligence (AI) to create this article.

## Results

The present study included 73 LTRs with a median age of 50 years (IQR: 38–59): females, 43 years (IQR: 29–56), and males, 50 years (IQR: 42–59). Male sex was dominant among the participants (sex ratio: male/female = 1/0.37) (Table 2). LTRs living in rural areas accounted for 6.9%, whereas 93.1% lived in urban areas (*p* < 0.001). People with low/intermediate levels of education were dominant compared with those with high levels of education (64.3% vs. 35.7%; *p* = 0.015). The number of participants living in households with four or more members was approximately twice that of individuals living in households with three or fewer members (34.3% vs. 65.7%; *p* = 0.007). Most recipients were nonsmokers (nonsmoking/smoking = 1/0.32, *p* < 0.001). Participants who did not drink 85% of their alcohol consumed alcohol (*p* < 0.001). The three most common causes of LT were hepatitis B virus infection (23.3%), alcohol-related liver disease (19.2%), and primary sclerosing cholangitis (16.4%). Laboratory parameters were within the reference range at follow-up (Table 2). The predominant immunosuppressive treatment was tacrolimus (57.5%) and the combination of tacrolimus + Mycophenolate mofetil (24.7%).

**Table 2.** Basic Characteristics of Liver Transplant Recipients Participating in the Present Research

Variable	LTRs (*n* = 73)
Sex, *n* (%)	
Female	20 (27.4)
Male	53 (72.6)
Age, years, mean ± SD	47.0 ± 14.0
Age groups, *n* (%)	
18–29	13 (17.8)
30–39	7 (9.5)
40–49	15 (20.5)
50–59	23 (31.7)
≥ 60	15 (20.5)
Place of residence, *n* (%)	
City	68 (93.1)
Village	5 (6.9)
Area of residence, part of the country, *n* (%)	
Northern Bulgaria	21 (28.7)
Southern Bulgaria	52 (71.3)
Level of education, *n* (%)	
Low/Intermediate	47 (64.3)
High	26 (35.7)
Members of household, *n* (%)	
Three or less	48 (65.7)
Four or more	25 (34.3)
Tobacco smoking after LTs, *n* (%)	
Yes	18 (24.6)
No	55 (75.4)
Frequency of alcohol consumption after LTs, *n* (%)	
2–3 times per week	2 (2.7)
2–3 times per month	9 (12.3)
Never	62 (85.0)
HEV testing, time after LTs, months, mean ± SD	82.6 ± 63.0
Reason for LTs, *n* (%)	
Hepatitis B virus (HBV)	17 (23.3)
Alcohol-related liver disease (ARLD)	14 (19.2)
Primary sclerosing cholangitis	12 (16.4)
Biliary atresia (BA)	7 (9.6)
Hepatocellular carcinoma (HCC)	5 (6.8)
Primary biliary cirrhosis	3 (4.1)
Wilson’s disease	3 (4.1)
Autoimmune hepatitis	2 (2.7)
HBV and Hepatitis D virus (HDV)	2 (2.7)
Secondary biliary cirrhosis	2 (2.7)
Another reason^a^	6 (8.2)
Laboratory parameters, median (IQR)	
ALT	28 (17–53)
AST	28 (22–45)
GGT	50 (22–138)
AP	88 (72–140)
Total bilirubin	15 (12–24)
Direct bilirubin	3 (2–6)
Total protein	73 (69–76)
Albumin	42 (40–45)
Creatinine	96 (83–116)
Immunosuppressive therapy, *n* (%)	
Tacrolimus	42 (57.5)
Tacrolimus + Mycophenolate mofetil	18 (24.7)
Tacrolimus + Everolimus	6 (8.2)
Everolimus	2 (2.7)
Ciclosporin	2 (2.7)
Mycophenolate mofetil	2 (2.7)
Tacrolimus + Everolimus + Mycophenolate mofetil	1 (1.4)

^a^ Another reason for LT: Budd-Chiari syndrome; congenital hepatic fibrosis; cryptogenic cirrhosis; epithelioid hemangioendothelioma involving multiple liver lesions; glycogen storage disease (GSD); hepatitis C virus (HCV).

Reference range: ALT/AST, 5–40 U/L; GGT, 10–50 U/L; AP, 64–300 U/L; total bilirubin, 5–21 µmol/L; direct bilirubin, 0–5 µmol/L; total protein, 66–87 g/L; albumin, 40–55 g/L; creatinine, 74–130 µmol/L.

ALT, alanine aminotransferase; AP, alkaline phosphatase; AST, aspartate aminotransferase; GGT, gamma-glutamyl transferase; HEV, hepatitis E virus; IQR, interquartile range; LTRs, liver transplant recipients; SD, standard deviation.

Twenty-five LTRs (34.2%) tested positive for anti-HEV IgG (Table 3). No anti-HEV IgM antibody-positive cases were observed. HEV seropositivity rates were higher in males compared than in females (37.7% vs. 25%), in persons over 60 years of age (40% vs. 32.7%), in those living in rural areas (40%), and in the southern part of the country (36.5%). Nonsmokers had almost three times higher, but not statistically significant, levels of anti-HEV IgG antibodies than smokers (40% vs. 16.6%; OR = 3.333). Logistic regression showed a statistically significant influence of educational level on HEV seropositivity; individuals with higher education were more likely to be HEV-positive (OR = 2.917, *p* = 0.038).

**Table 3.** Logistic Regression for Association Between HEV Seropositivity and Different Basic Characteristics

Characteristics	Total LTRs, *n*	HEV positive, *n* (%)	Binary logistic regression		
			OR	95% CI	*p* Value
Sex, *n* (%)					
Female	20	5 (25.0)	1.000		
Male	53	20 (37.7)	1.818	0.573–5.768	0.310
Age, *n* (%)					
<60 years	58	19 (32.7)	1.000		
≥60 years	15	6 (40.0)	1.368	0.425–4.407	0.599
Area of residence, part of the country, *n* (%)					
Northern Bulgaria	21	6 (28.5)	1.000		
Southern Bulgaria	52	19 (36.5)	1.439	0.478–4.334	0.517
Level of education, *n* (%)					
Low/intermediate	47	12 (25.5)	1.000		
High	26	13 (50.0)	2.917	1.062–8.011	0.038
Members of household, *n* (%)					
Three or less	48	14 (29.1)	1.000		
Four or more	25	11 (44.0)	1.908	0.698–5.215	0.208
Tobacco smoking, *n* (%)					
Yes	18	3 (16.6)	1.000		
No	55	22 (40.0)	3.333	0.863–12.882	0.081
Alcohol consumption, *n* (%)					
No	62	20 (32.2)	1.000		
Yes	11	5 (45.4)	1.750	0.477–6.426	0.399

OR, odds ratio; CI, confidence interval.

Risk factors associated with HEV infection are shown in Table 4. We observed higher HEV-positive results in individuals who consumed pork meat (95% confidence interval [CI]: 0.562–6.768%), meat from wild animals (95% CI: 0.802–9.241%), meat cooked “medium rare/medium” (95% CI: 0.863–8.804%), and seafood (95% CI: 0.413–2.993%). Furthermore, higher HEV seropositivity was found among LTRs who stored food in the basement (95% CI: 0.737–10.004%) and those who butchered or skinned domestic pigs or wild animals (95% CI: 0.308–7.297%). No statistically significant association was observed for any of the risk factors (*p* > 0.05).

**Table 4.** Logistic Regression for Association Between HEV Seropositivity and Associated Risk Factors

Risk factors	Total LTRs, *n*	HEV positive, *n* (%)	Binary logistic regression		
			OR	95% CI	*p* Value
Consumption of pork meat and pork products (sausage, salami, etc.), *n* (%)					
No	17	4 (23.5)	1.000		
Yes	56	21 (37.5)	1.950	0.562–6.768	0.293
Consumption of meat and meat products from wild animals, *n* (%)					
No	60	18 (30.0)	1.000		
Yes	13	7 (53.8)	2.722	0.802–9.241	0.108
Consumption of meat and meat products cooked “medium rare/medium,” *n* (%)					
No	58	17 (29.3)	1.000		
Yes	15	8 (53.3)	2.756	0.863–8.804	0.087
Consumption of seafood, *n* (%)					
No	45	15 (33.3)	1.000		
Yes	28	10 (35.7)	1.111	0.413–2.993	0.835
Drinking water use, *n* (%)					
Personally bottled water from a free water source	8	2 (25.0)	1.000		
Bottled water by the industry	50	15 (30.0)	1.452	0.261–8.066	0.670
Public water supply	28	11 (39.2)	2.182	0.346–13.756	0.406
Type of sewage, *n* (%)					
Septic tank	8	2 (25.0)	1.000		
Public sewers	65	23 (35.3)	1.643	0.306–8.807	0.562
One kitchen board for cooking, *n* (%)					
No	34	11 (32.3)	1.000		
Yes	39	14 (35.8)	1.171	0.443–3.094	0.750
Pets (dog or cat), *n* (%)					
No	45	14 (31.1)	1.000		
Yes	28	11 (39.2)	1.433	0.534–3.843	0.475
Rodent control in the basement, *n* (%)					
Yes	11	3 (27.2)	1.000		
No	62	22 (35.4)	1.467	0.353–6.100	0.598
Storage of food in the basement, *n* (%)					
No	62	19 (30.6)	1.000		
Yes	11	6 (54.5)	2.716	0.737–10.004	0.133
Contact with domestic pigs, *n* (%)					
No	56	19 (33.9)	1.000		
Yes	17	6 (35.2)	1.062	0.340–3.315	0.917
Butchering or skinning on domestic pigs or wild animals, *n* (%)					
No	66	22 (33.3)	1.000		
Yes	7	3 (42.8)	1.500	0.308–7.297	0.615

## Discussion

HEV infection can be a serious medical problem in SOT recipients. HEV seropositivity in LTRs differed significantly between different countries and authors: Japan, 2.9% (Inagaki et al., 2015); the Netherlands, 3.0% (Haagsma et al., 2009); Spain, 7.4% (Riveiro-Barciela et al., 2014); Sweden, 13.0% (Frankal et al., 2022); Iran, 15.6% (Chorami et al., 2021); Türkiye, 20.7% (Celik et al., 2021); and Croatia, 24.4% (Mrzljak et al., 2019). The overall HEV seroprevalence in the general population was similar in Spain, 2.17% (Fogeda et al., 2012); Italy, 2.9% (Vulcano et al., 2007); Japan, 5.3% (Takahashi et al., 2010), and 7.0% (Yoshida et al., 2024); the Republic of Korea, 5.9% (Yoon et al., 2014); Slovakia, 7.2% (Halanova et al., 2018); Norway, 11.4% (Olsoy et al., 2019); Taiwan, 11.5% (Lee et al., 2013); Portugal, 16.3% (Nascimento et al., 2018); Saudi Arabia, 23.8% (El-Daly et al., 2023); and the Netherlands, 28.7% (van Gageldonk-Lafeber et al., 2017). Our results (34.2%) were similar to data from Croatia (24.4%; LTRs) (Mrzljak et al., 2019) and the Netherlands (28.7%; general population) (van Gageldonk-Lafeber et al., 2017). A comparison between HEV seropositivity in LTRs and the general population revealed no significant differences between the two groups.

The risk factors for HEV seroprevalence in LTRs have varied among studies. Mrzljak et al. (2019) reported as risk factors for higher HEV IgG positive results 1-year increase of age (*p* = 0.004), male sex (*p* < 0.001), and sewage system type “septic tank” (*p* = 0.005). Turkish authors found that the age (*p* = 0.007), living donor (*p* = 0.049), tap water (*p* = 0.034), and other place of birth different from Eastern and Southeastern Anatolia (*p* < 0.001) were associated risk factors for HEV infection in LTRs (Celik et al., 2021). Frankal et al. (2022) did not establish any risk factors among 109 LTRs from Sahlgrenska University Hospital in Gothenburg, Sweden. Spanish scientists reported that factors independently associated with anti-HEV seroprevalence were liver cirrhosis (OR = 7.6; *p* < 0.001), orthotopic liver transplantation (OR = 3.1; *p* < 0.001), and HIV infection (OR = 2.4; *p* = 0.006) obtained by multivariate analysis (Riveiro-Barciela et al., 2014).

Similar risk factors have been reported for higher HEV seropositivity in the general population. Olsoy et al. (2019) reported for the following potential risk factors among 1800 persons from Northern Norway: increasing age (OR = 1.036 per year; *p* < 0.001) and higher education (OR = 2.167; *p* < 0.001). Dutch authors found male sex and low level of education such as variables associated with an increased risk for HEV seroprevalence in 2494 adults (van Gageldonk-Lafeber et al., 2017). Toyoda et al. (2008) presented the consumption of undercooked or raw boar meat as a risk factor for higher HEV seropositivity among the general population of Okinawa, Kyushu, Japan. Korean scientists announced that HEV seroprevalence was significantly higher among agricultural, forestry, and fishery workers, as well as in groups with males, older age, low education level, and living in rural areas (Yoon et al., 2014). Lee et al. (2013) reported that swine farmers had a 3.46-fold increased risk (95% CI: 1.91–6.27; *p* < 0.0001) for being seropositive for anti-HEV IgG compared with the general population established by the logistic regression analysis.

In the present study, we found a high HEV IgG seroprevalence (34.2%). The potential reasons for this could be many and varied. First, there are high levels of HEV seropositivity in different animal and human populations in our country. High levels of HEV-positive results were observed in Bulgaria among goats, 24.4% (Tsachev et al., 2023); sheep, 32.2% (Tsachev et al., 2023); swine from farrow-to-finish farms, 36.0% (Tsachev et al., 2021a); wild boars, 40.8% (Tsachev et al., 2021b); domestic pigs, 60.0% (Takova et al., 2020); and East Balkan swine, 82.5% (Tsachev et al., 2020). In addition, high HEV seropositivity was observed in Bulgarian patients with Guillain-Barre syndrome, 24.5% (Golkocheva-Markova et al., 2023); blood donors, 25.9% (Baymakova et al., 2021); general hunters, 48.7% (Baymakova et al., 2021); and hunters of wild boars, 51.6% (Baymakova et al., 2021). Second, in the present study, the male sex was predominant (72.6%), and it is well known from the scientific literature that males are more often affected by HEV (Inagaki et al., 2015; ECDC, 2017; Frankal et al., 2022). Third, the majority of the participants lived in Southern Bulgaria (71.3%), which corresponds to the higher HEV seropositivity found in our previous research in this part of the country (Baymakova et al., 2021). Fourth, an important potential reason for our high HEV-positive results (34.2%) could be the high percentage of LTRs who consumed pork meat and pork products (76.7%). In this regard, many authors have reported that the consumption of pork meat and pork products is a risk factor for infection with *Paslahepevirus* genus (HEV gt 3 and HEV gt 4) (Kmush et al., 2015; Mooij et al., 2018; Capai et al., 2019). Fifth, another possible reason for the high HEV seropositivity could be the low rodent control rate—84.9% of participants in the current study did not perform rodent control in their basements. These data support the hypothesis that some HEV cases are associated with contact with rodents or rodent-contaminated food. This hypothesis is based on data from a large number of cases of rat HEV (*Rocahepevirus ratti*) in humans in recent years (Andonov et al., 2019; Sridhar et al., 2021; Rivero-Juarez et al., 2022).

Interestingly, we found two times higher HEV seropositivity in individuals with a high level of education than in those with low or intermediate levels of education (50% vs. 25.5%; OR = 2.917; *p* = 0.038). After a careful review of our data, there are several potential reasons for this. First, the male sex was dominant (76.9%) among the highly educated LTRs. Second, the majority of the participants with higher education were residents of Southern Bulgaria (76.9%). Third, most LTRs with higher education regularly consume pork meat and pork products (80.7%). These three potential factors dominated data analysis. Based on a review of the scientific literature (Inagaki et al., 2015; Kmush et al., 2015; ECDC, 2017; Mooij et al., 2018; Capai et al., 2019; Baymakova et al., 2021; Frankal et al., 2022), we hypothesized that these may be potential reasons for the higher HEV seropositivity among our LTRs with a high level of education.

The applied diagnostic test may have influenced the results. Indirect diagnostic tests are based on the detection of specific antibodies. Various commercial assays (ELISA tests) are used in daily practice. Several publications compared their parameters and estimated high specificity, but the sensitivity varied (Pas et al., 2013; Avellon et al., 2015; Norder et al., 2016). Pas et al. (2013) summarized that the analyzed ELISA tests (selected commercially available) could be applied clinically to search for both HEV gt 1 and HEV gt 3. The analysis of Norder et al. (2016) found a wide variation in anti-HEV detection sensitivities (42–96%). Also, the highest concordance in anti-HEV IgG reactivity was established in the Dia.Pro and Axiom (corresponding to Wantai) assays (Norder et al., 2016). Furthermore, the variations in sensitivity might be influenced by the technical basis, the antigens used, and the cutoff definition (Avellon et al., 2015). Despite done improvements in serological assays, no gold standard exists for HEV infection, and none has been approved by the U.S. Food and Drug Administration. There are many problems and disputes with their concordance, standardization, and validation (Zhao and Wang, 2016). In this regard, WHO reference reagent for antibodies to HEV is applied for calibration (Ferguson et al., 2002). In addition, a review of the scientific literature showed that the three most commonly applied serological tests were Wantai HEV ELISA kits (Beijing, China), Dia.Pro HEV ELISA kits (Milan, Italy), and *recom*Well HEV ELISA kits (Mikrogen GmbH, Neuried, Germany) (Pas et al., 2012; Rossi-Tamisier et al., 2013; Buffaz et al., 2014; Koning et al., 2015; Thom et al., 2018; Mrzljak et al., 2019; Chorami et al., 2021; Frankal et al., 2022; Öğüt et al., 2022; Samala et al., 2022). We performed Dia.Pro HEV ELISA tests (Milan, Italy); therefore, we thought that our results were correct and presented the real situation.

The main limitation of the present study was the small number of participants. The leading cause for this was the small number of LTs that had been performed in Bulgaria (1.68 LTs rate per million population (*pmp*), 2013–2022) (GODT, 2024) compared with other countries in our region during the same period—Croatia (27.84 LTs *pmp*), Türkiye (17.67 LTs *pmp*), and Slovenia (11.47 LTs *pmp*) (GODT, 2024). Additionally, molecular analysis of HEV (HEV RNA testing) has not been performed. Despite these limitations, the present study had several merits. To the best of our knowledge, this is the first seroepidemiological study on HEV among LTRs in Bulgaria. In addition, we investigated and analyzed the risk factors associated with HEV infection among the LTRs.

## Conclusion

The current survey showed widespread HEV among immunosuppressed individuals in Bulgaria (34.2%), which corresponds to the data from other studies (Bruni et al., 2018; Tsachev et al., 2020, 2021a, 2021b, 2023; Baymakova et al., 2021; Golkocheva-Markova et al., 2022, 2023; Kevorkyan et al., 2023). The statistically significant factor is “high level of education” (OR = 2.917; *p* = 0.038), for which several potential reasons could influence and more detailed future studies should help to explain this. In conclusion, a national information campaign on HEV infection (distribution, routes of transmission, and clinical symptoms) in our country is warranted as well as adequate control measures and surveillance for HEV infection among high-risk groups.

## Data Availability

Data will be made available upon reasonable request by other researchers. Scientists with other inquiries or collaboration proposals may contact M.P.B.—the principal investigator in charge of setting up the present research.
