# Supplementary material for: First Insight into the Seroprevalence of Hepatitis E Virus and Associated Risk Factors Among Liver Transplant Recipients from Bulgaria
Source: Vector Borne Zoonotic Dis. 2025 Apr 2;25(4):303–13. doi: 10.1089/vbz.2024.0101 (PMC12995555; doi:10.1089/vbz.2024.0101)
Supplement: Supplementary Table S1 [file vbz.2024.0101_supplementarytables1.docx]

**Supplemental File**

**Table S1.** Paper-Based Survey Questionnaire “Hepatitis E Virus (HEV) and Associated Risk Factors Among Liver Transplant Recipients (LTRs) from Bulgaria”

| **Part 1 (completed by participant)**  (1.) Sex: (a) Male; (b) Female.  (2.) Age (years).  (3.) Place of residence: (a) City; (b) Village.  (4.) Area of residence, part of the country: (a) Northern Bulgaria; (b) Southern Bulgaria.  (5.) Level of education: (a) Low/Intermediate; (b) High.  (6.) Members of household: (a) Three or less; (b) Four or more.  (7.) Tobacco smoking after LTs: (a) Yes; (b) No.  (8.) Frequency of alcohol consumption after LTs: (a) 2-3 times per week; (b) 2–3 times per month; (c) Never.  (9.) Consumption of pork meat and pork products (sausage, salami, etc.): (a) Yes; (b) No.  (10.) Consumption of meat and meat products from wild animals: (a) Yes; (b) No.  (11.) Consumption of meat and meat products cooked “medium rare/medium”: (a) Yes; (b) No.  (12.) Consumption of seafood: (a) Yes; (b) No.  (13.) Drinking water use: (a) Personally bottled water from a free water source; (b) Bottled water by the industry; (c) Public water supply.  (14.) Type of sewage: (a) Septic tank; (b) Public sewers.  (15.) One kitchen board for cooking: (a) Yes; (b) No.  (16.) Pets (dog or cat): (a) Yes; (b) No.  (17.) Rodent control in the basement: (a) Yes; (b) No.  (18.) Storage of food in the basement: (a) Yes; (b) No.  (19.) Contact with domestic pigs: (a) Yes; (b) No.  (20.) Butchering or skinning on domestic pigs or wild animals: (a) Yes; (b) No. |
| --- |
| **Part 2 (completed by physician)**  (21.) HEV testing, time after LTs (months).  (22.) Reason for LTs.  (23.) Laboratory parameters.  (24.) Immunosuppressive therapy.  (25.) HEV IgG ELISA result: (a) Positive; (b) Negative.  (26.) HEV IgM ELISA result: (a) Positive; (b) Negative. |
